# Precision nephrotoxicity testing using 3D in vitro models

**DOI:** 10.1186/s13578-023-01187-0

**Published:** 2023-12-21

**Authors:** Pengfei Yu, Hainan Zhu, Carol Christine Bosholm, Daniella Beiner, Zhongping Duan, Avinash K. Shetty, Steve S. Mou, Philip Adam Kramer, Luis F. Barroso, Hongbing Liu, Kun Cheng, Michael Ihnat, Matthew A. Gorris, Joseph A. Aloi, Jobira A. Woldemichael, Anthony Bleyer, Yuanyuan Zhang

**Affiliations:** 1 Wake Forest Institute for Regenerative Medicine, Wake Forest University Health Sciences, Winston-Salem, NC USA; 2 The Fourth Department of Liver Disease, Beijing You An Hospital, Capital Medical University, Beijing, China; 3 Department of Pediatrics, Wake Forest University School of Medicine, Winston-Salem, NC USA; 4 Department of Anesthesiology and Pediatrics, Wake Forest University School of Medicine, Winston-Salem, NC USA; 5 Department of Internal Medicine, Section on Gerontology and Geriatrics, Wake Forest University School of Medicine, Winston-Salem, NC USA; 6 Internal Medicine/Infectious Diseases, Wake Forest University Health Sciences, Winston-Salem, NC USA; 7 Department of Pediatrics and The Tulane Hypertension and Renal Center of Excellence, Tulane University School of Medicine, Tulane Avenue, New Orleans, LA USA; 8 Division of Pharmacology and Pharmaceutical Sciences, School of Pharmacy, University of Missouri-Kansas City, 2464 Charlotte Street, Kansas City, MO 64108 USA; 9 Department of Pharmaceutical Sciences, University of Oklahoma College of Pharmacy, University of Oklahoma Health Sciences Center, Oklahoma City, OK USA; 10 Division of Endocrinology and Metabolism at Wake Forest Baptist Health, Winston-Salem, NC USA; 11 Division of Nephrology, Wake Forest University Health Sciences, Winston-Salem, NC USA

**Keywords:** Precision medicine, Nephrotoxicity, Drug toxicity testing, 3D culture model, Human primary renal cells

## Abstract

Nephrotoxicity is a significant concern during the development of new drugs or when assessing the safety of chemicals in consumer products. Traditional methods for testing nephrotoxicity involve animal models or 2D in vitro cell cultures, the latter of which lack the complexity and functionality of the human kidney. 3D in vitro models are created by culturing human primary kidney cells derived from urine in a 3D microenvironment that mimics the fluid shear stresses of the kidney. Thus, 3D in vitro models provide more accurate and reliable predictions of human nephrotoxicity compared to existing 2D models. In this review, we focus on precision nephrotoxicity testing using 3D in vitro models with human autologous urine-derived kidney cells as a promising approach for evaluating drug safety.

## Background of in vitro drug-induced nephrotoxicity assays

Drug-induced nephrotoxicity refers to kidney damage or dysfunction caused by drugs or their metabolites [1]. In the inpatient setting, about 20% to 60% of cases of acute kidney injury (i.e., abrupt loss of renal excretory function) have been attributed to drug-induced nephrotoxicity in both adults and children, leading to significant morbidity and mortality [2–4]. Novel biomarkers have been proposed to aid in the identification of acute kidney injury, but their precise role has not yet been evaluated [3]. Thus, an optimal in vitro model to inform early and more sensitive detection of renal toxicity is urgently desired. An ideal model would be specific enough to identify the sites of renal cell injury and relate to the phenotype of the injury.

## Precision nephrotoxicity testing

Functional precision medicine using patient-derived assays [5] is a relatively new concept that involves the use of advanced technologies and techniques to develop personalized and precise approaches for predicting, diagnosing, and treating various diseases. This approach recognizes that individual patients may respond differently to the same drug due to variations in genetic makeup, environmental factors and other variables. Traditional approaches to drug-induced kidney injury often rely on clinical symptoms and laboratory tests that may not be sensitive or specific enough to detect subtle changes in kidney function. Precision nephrotoxicity testing aims to overcome these limitations by leveraging cutting-edge technologies such as genomics, proteomics, metabolomics and imaging to develop more accurate and sensitive biomarkers. In this review, we discuss precision nephrotoxicity testing using primary human renal cells in 3D in vitro models as a method for predicting drug-induced kidney injury [6–9].

Precision nephrotoxicity assessment requires two key components: 3D culture systems and human primary renal cells. These two components are crucial for creating a more precise and relevant model to evaluate the potential toxicity of drugs and chemicals to the kidneys. 3D culture systems can provide a more physiologically relevant environment, while human primary renal cells are the most relevant cell type to the human kidney. By combining these two components, researchers can better evaluate drug safety and reduce the risk of adverse effects on patients.

Traditional in vitro methods of testing for nephrotoxicity rely on 2D cell cultures, which have limitations in accurately predicting drug-induced nephrotoxicity in humans. 3D in vitro models, such as spheroids, organoids, and microtissues [10], have emerged as a promising alternative to overcome these limitations and improve the accuracy of drug testing. This is because 3D models can better recapitulate the cellular and structural complexity of human kidneys, allowing for more accurate cell–cell and cell-extracellular matrix interactions.

3D models more closely mirror cellular architecture [9, 11], gene expression patterns [12], oxygen and nutrient diffusion dynamics [13], than 2D culture models. Drugs known to affect microarchitecture, mitochondrial function, and alter gene expression, (i.e., oxygen consumption rate/extracellular acidification rate [OCR/ECAR], mitochondrial membrane potential [MMP], adenosine triphosphate [ATP] production, complex I-V expression and activity, mitochondrial morphology, DNA content-Mitochondrial [mtDNA] content, apoptosis, and reactive oxygen species [ROS]) demonstrate better physiological toxicity in 3D vs 2D models [8, 9]. These provide a more realistic representation of in vivo physiology and better predict drug toxicity compared to 2D cultures. In addition, 3D models can reduce the need for animal testing, which is important from ethical and financial considerations [14].

For 3D models, the choice of cell type is critical. Human primary renal cells are preferable to cell lines and animal renal cells because they better replicate the physiology of human kidneys [15]. Most cell lines are immortalized and demonstrate elevated metabolic activity, altered proliferation migration profiles, aberrant cell death pathways and gene expression patterns, and toxicity resistance or susceptibility not reflective of normal kidney tissues. However, human primary renal cells are difficult to obtain and culture and often do not represent the heterogeneous cell types present in kidney tissue.

We are the first to demonstrate the existence of renal progenitor cells in urine, which we have named urine-derived stem cells (USCs) [16, 17]. These cells are readily accessible, easy to isolate and expand, and have the potential to differentiate into multiple renal cell types (i.e., podocytes, renal tube epithelial cells [6] and endothelial cells [18]). They also exhibit paracrine effects and possess low immunogenicity, making them suitable for tissue repair. USCs form functional tubular structures in 3D cultures. These properties make USCs a valuable cell source for the development of 3D in vitro models for evaluating drug-induced nephrotoxicity [6, 7] and renal mitochondrial toxicity [8, 9]. This review specifically focuses on precision nephrotoxicity testing using human renal cells, particularly USCs, in 3D in vitro models. This approach has the potential to significantly reduce the number of animals used for toxicity testing and improve the efficiency of drug development. Further research is needed to optimize and validate these models for routine use in preclinical drug screening.

## Comparison of 2D and 3D cultures in renal toxicity testing

Two-dimensional (2D) cell culture is a widely used method in renal toxicity assessment, offering several advantages. These include being cost-effective, easy to use, high-throughput, reproducible, compatible with imaging techniques and having established protocols. These advantages make 2D culture an initial test option for renal toxicity testing, allowing for reliable and efficient screening of potentially toxic compounds. However, 3D in vitro models can better replicate the structural and functional features of the human kidney. They allow for the examination of drug metabolism and transport within the kidney and can integrate different cell types to simulate the cellular heterogeneity of the kidney (Table 1).

**Table 1:** Advantages and disadvantages of 3D compared to 2D models for nephrotoxicity testing

	2D culture	3D culture
Advantages	• Cost-effective • High-throughput • Accessibility • Short-term assay (2-week)	• Increased physiological relevance • Better recapitulation of tissue architecture, cell–cell communications • Bridge gaps between in vitro and in vivo •> 4-week assay
Disadvantages	• Lack of physiological relevance • Limited cell–cell interactions • Altered metabolism • Cell line dependency	• Technical complexity • Cost • Variability • Limited throughput

Several studies have demonstrated that 3D cultures are more effective than 2D cultures for studying drug-induced nephrotoxicity. For example, cultured proximal tubule cells were used in both 2D and 3D conditions and exposed to gentamicin, a nephrotoxic drug. Gentamicin-induced cell death was higher in 2D cultures compared to 3D cultures, indicating that 3D cultures better mimic the in vivo response to drug toxicity [19]. Similarly, King et al. [20] compared the response of human kidney cells to cisplatin, a commonly used nephrotoxic chemotherapeutic drug, in 2D and 3D cultures. The study found that 3D cultures better preserved cell viability and morphology than 2D cultures and provided more accurate information about cisplatin-induced-nephrotoxicity. In another study, Vormann et al. [21]. used a microfluidic 3D culture system to evaluate the nephrotoxicity of four model nephrotoxic drugs (cisplatin, tenofovir, tobramycin and cyclosporin A). The authors found that the microfluidic 3D system better reflected the in vivo response to the drug compared to 2D cultures, suggesting that 3D cultures can provide a more accurate assessment of drug-induced nephrotoxicity.

3D models have several advantages over 2D models for renal toxicity testing, such as the ability to better mimic the complex 3D environment of the kidney, allowing for more accurate predictions of drug efficacy and toxicity. Particularly, 3D culture tissues better represent in vivo cell–cell communication, cell–matrix interaction, physiologically relevant oxygen and nutrient diffusion dynamics, though this is size dependent as larger cultures can become anoxic [13]. However, 3D models are more technically complex and expensive than 2D models. The creation and maintenance of 3D models require specialized equipment and expertise, making them less accessible for some researchers. Additionally, the higher cost of 3D models can be a barrier for some laboratories. Despite these challenges, the benefits of 3D models in renal toxicity testing make them a valuable tool, and their use is becoming increasingly common in the field.

## 3D cultures for nephrotoxicity testing

There are various types of 3D cultures used for drug-induced nephrotoxicity testing, including spheroids, organoids, cell-scaffold constructs, microfluidic devices, bioprinted structures and co-culture models.

Several 3D culture models have been developed for drug-induced renal testing (Table 2).

**Table 2:** 3D in vitro cell culture models for drug-induced nephrotoxicity testing

3D model	Cell Types	Cell number	Drugs and dose	Treatment time
Spheroids	Rptec-tert1	6.5*10^5^ cells/mL	Cadmium chloride (55 µM) Cisplatin (50 µM)	48 h
Organoids	Pluripotent stem cell	500–800 organoids/well	Cisplatin (50 µM)	7 days
Cell-scaffold construct	Urine-derived stem cells	5 × 10^5^ cells	Zalcitabine (10 μM)	6 weeks
Microfluidic devices	Proximal tubule cells, microvascular endothelial cells	7.5 × 10^4^ cells	Cisplatin (400 μM) Cyclosporine (450 μM)	24 h
Bioprinting technology	Renal epithelial cell	34 × 10^6^ cells	Cisplatin (100 μM)	48 h
Co-culture Models	renal proximal tubular epithelial cells, peritubular capillary endothelial cells	5 × 10^5^/mL	Cisplatin (40 μmol/L), Gentamycin (40 μmol/L), Cyclosporin A (40 μmol/L)	1, 4, and 7 days

Spheroids: Spheroids are 3D aggregates of single renal cells, such as human renal tubular epithelial cells, or undifferentiated renal stem cells (e.g. USCs [9]). They can be generated using different techniques, such as hanging-drop, centrifugation, or non-adherent surfaces [22].

Organoids: Renal organoids, including those derived from human pluripotent stem cells or kidney-specific progenitor cells, offer a 3D model that can mimic the cellular composition and functional characteristics of the kidney. These organoids provide advantages such as recapitulating kidney development, cellular diversity, disease modeling potential, and the ability to perform high-throughput screening. However, challenges exist in terms of their immaturity, variability, and complexity. Ongoing progress involves improving organoid maturation, enhancing vascularization, and integrating organoids with other tissues to study inter-organ interactions [23, 24].

Cell-scaffold construct: Seeding cells in a polymer fiber scaffold with high porous micro-contracture is a common approach for creating 3D culture models for nephrotoxicity testing. Porous scaffolds provide a 3D environment that mimics the microarchitecture of the kidney and allows for the growth and differentiation of kidney cells. The porous scaffold can be made from various materials, such as polycaprolactone (PCL), poly (lactic-co-glycolic acid) (PLGA), silk fiber matrix or collagen. The scaffold provides structural support for the cells, enabling them to form complex networks and functional units, such as tubules and glomeruli [19, 25–27].

Microfluidic devices: Microfluidic devices are small-scale systems that manipulate fluids and particles in a precise and controlled manner for various applications, including chemical analysis, drug discovery, and biological research. Kidney-on-a-chip systems are microfluidic platforms designed to replicate the structural and functional properties of the kidney. These models offer controlled environments for studying nephrotoxicity, allowing the assessment of cellular responses to drugs or toxins. Advantages include the ability to simulate physiological conditions, replicate mechanical and biochemical cues, and perform real-time monitoring. Challenges lie in the complexity of fabrication and operation. Future directions involve improving chip design, enhancing functionality, and integrating advanced sensing and imaging technologies [21, 28–30].

Bio-printing technology: This approach has been increasingly used to fabricate 3D tissue models for in vitro toxicity testing (see Part 8). 3D bioprinting enables the fabrication of complex kidney tissue constructs using a layer-by-layer approach. This technique combines renal cells, biomaterials, and growth factors to generate tissue-like structures. Bioprinted kidney tissue allows for control over spatial organization and cell distribution, enabling the recreation of native kidney architecture. Advantages include the ability to mimic tissue complexity, precise control over cell composition, and the potential for personalized medicine. Challenges involve optimizing bioprinting techniques, achieving vascularization, and scaling up production. Future directions involve improving cell viability and function, enhancing vascularization techniques, and advancing bioprinting technology. Bioprinted structures are advantageous since they are able to mimic the complex 3D renal architecture in a precise and highly reproducible manner [31–35].

Co-culture Models: Co-culture models involve the interaction of different cell types within a 3D environment to mimic the cellular composition and interactions of the kidney. These models can be created by culturing renal cells with other relevant cell types, such as endothelial cells or immune cells. Co-culture models offer advantages in studying cell–cell interactions and their influence on nephrotoxicity. Challenges include establishing and maintaining proper cell ratios, optimizing culture conditions, and interpreting complex interactions. Future directions involve developing more sophisticated co-culture models that replicate specific renal compartments and exploring the role of immune cells in nephrotoxicity [20, 36].

Applications of 3D In Vitro Models of Nephrotoxicity include: (1) Early safety assessment of drugs and chemicals: 3D models enable the evaluation of nephrotoxicity at an early stage, reducing the reliance on animal testing and improving prediction accuracy [37]. (2) Mechanistic studies: These models help elucidate the underlying mechanisms of nephrotoxicity, including cellular responses, oxidative stress, inflammation, and tissue damage [38]. (3) Disease modeling: 3D models offer the ability to study genetic kidney diseases, such as polycystic kidney disease or Alport syndrome, providing insights into disease mechanisms and potential therapeutic targets [39]. (4) Personalized medicine: Patient-specific induced pluripotent stem cell-derived organoids allow the study of individual patient responses to nephrotoxic compounds, facilitating personalized treatment strategies [40].

Future Directions: (1) Maturation and functional improvement: Enhancing the maturity and functionality of 3D models to better resemble adult kidney tissue and accurately replicate kidney function. (2) Vascularization: Developing techniques to incorporate vascular networks within 3D models, allowing better nutrient delivery, waste removal, and mimicry of the renal vasculature. (3) Integration of multiple organ systems: Establishing multi-organ models that combine kidney models with other organ models to study systemic drug toxicity, drug-drug interactions, and disease modeling. (4) High-throughput automation: Developing automated systems for large-scale production, characterization, and screening of 3D models to improve efficiency and reproducibility. (5) Computational modeling: Combining experimental data with computational models to simulate nephrotoxicity and predict toxic outcomes more accurately.

In summary, 3D in vitro models of nephrotoxicity, including renal organoids, kidney-on-a-chip, 3D bioprinted kidney tissue, and co-culture models, offer unique advantages and challenges. These models have applications in drug screening, mechanistic studies, disease modeling, and personalized medicine. Future directions involve improving model maturity and functionality, vascularization, integration of multiple organ systems, high-throughput automation, and computational modeling to enhance the utility of these models in nephrotoxicity research and drug development.

## Different types of cells for 3D models

Different types of cells can be used to create 3D models, including human primary cells, animal renal cells, and cell lines (Table 3).

**Table 3:** Comparison of human primary cells and cell lines

	Cell lines	Human primary cells	iPSC-derived cells
	HK-2, RPTEC/TERT1, LLC-PK1	Proximal tubular epithelial cells, Mixed renal tubule cells, Podocytes	All renal cell types
Advantages	• Reproducibility • Homogeneity • Availability	• High physiological relevance • Greater genetic diversity • Reduced culture artifacts • Improved translational potential	• Patient-specific models • Recapitulation of renal development • Cellular diversity • High-throughput screening
Disadvantages	• Lack of physiological relevance • Limited diversity • Genetic drift • Cell culture artifacts	• Limited availability • Limited lifespan • Heterogeneity • High cost	• Immaturity • Variability • Complexity

*HK-2* human proximal tubule epithelial cells, *RPTEC/TERT1* human renal proximal tubule epithelial cells/human telomerase reverse transcriptase, *LLC-PK1* pig kidney epithelial cells

*Human primary cells*: Primary human renal cells offer a more accurate representation of the kidney's physiology compared to renal tubular cell lines. These cells, such as proximal tubular epithelial cells, can be isolated from human kidney tissues and reflect the kidney's cellular heterogeneity and complexity [30, 41]. However, human podocytes are less commonly used due to challenges in their isolation and expansion in culture. While primary human cell-derived 3D models provide a physiologically relevant representation of renal cell types, there are important considerations. It’s crucial to acknowledge the limitations and make efforts to incorporate cell type heterogeneity in the right proportions. The kidney consists of various cell types, including podocytes, endothelial cells, mesangial cells, nephron tubule segments (from proximal to distal), and collecting duct cell types, each with distinct characteristics and drug responses [42]. To better mimic the complexity of the kidney, all renal cell types in a relative normal ratio should ideally be used in these models [43–45].

Comparative studies among different 3D models, including those with multiple renal cell types, are necessary to assess which models best capture renal cell type heterogeneity. Evaluation could encompass aspects like cellular morphology, gene expression profiles, functional characteristics, and responses to nephrotoxic compounds [46–48]. In future research on 3D in vitro models of nephrotoxicity, a comprehensive analysis may focus on the specific cell types included in the models, their relevance to renal transport physiology, and their performance relative to other 3D models with multiple renal cell types. These approaches will enhance our understanding of how well different models represent the cellular heterogeneity of the kidney and their suitability for nephrotoxicity studies.

*Animal primary cells*: Renal cells from animals, such as rats, can be isolated from animal kidneys and can provide a more accessible alternative to primary human cells [26, 49, 50]. However, animal renal cells may differ significantly from human renal cells in their metabolic pathways and responses to drugs.

*Cell lines*: Several kidney-derived cell lines, such as HK-2, RPTEC/TERT1, and LLC-PK1 provide a convenient and cost-effective alternative to primary human cells. However, they are genetically homogenous and may not precisely replicate the physiological characteristics of the human kidney [51–54].

Human iPSC-Derived Organoids: Human induced pluripotent stem cell (iPSC)-derived organoids, also known as 3D culture model of renal cells, represent an advancement in nephrotoxicity studies [55]. These 3D spherical structures are generated by differentiating human pluripotent stem cells into kidney-specific cell types, closely resembling the kidney tissue. They can recapitulate various aspects of kidney composition and structure, including the formation of nephrons and interactions between different cell types [47, 56, 57]. They offer several advantages [45, 58, 59]: (a) they recapitulate renal development: Organoids mimic key features of kidney development, providing insights into developmental processes and disease mechanisms. (b) Cellular diversity: Organoids contain multiple cell types, including proximal tubules, distal tubules, and podocytes, enabling the study of nephrotoxicity in a more physiologically relevant context. (c) Disease modeling: They can be derived from patient-specific iPSCs, allowing the study of genetic kidney diseases and personalized medicine approaches. (d) High-throughput screening: Organoids can be generated in large numbers, facilitating high-throughput screening of nephrotoxic compounds.

However, it’s important to note that human iPSC-derived organoids have their own challenges [55, 60–62]: (a) immaturity: Organoids do not fully resemble mature adult kidneys and may exhibit fetal or neonatal characteristics. (b) Variability: There can be variability between different organoid batches or between different iPSC lines, leading to challenges in standardization and reproducibility. (c) Complexity: The complex nature of organoids makes their characterization and analysis challenging. Future directions in this field include efforts to enhance organoid maturation, incorporate vascular networks for improved functionality, establish multi-organoid systems to study systemic drug toxicity, and develop automated systems for scalability and reproducibility.

In summary, a variety of cell types, including human primary cells, animal primary cells, and cell lines, have been utilized in the establishment of 3D models for assessing drug-induced nephrotoxicity. While animal primary cells and cell lines present more accessible and cost-effective alternatives, primary human cells offer a closer resemblance to human kidney physiology. However, it is difficult to obtain sufficient primary human renal cells for drug screening. Thus, human induced pluripotent stem cell (iPSC)-derived organoids show promise as a platform for precision medicine in nephrotoxicity research. They possess advantages such as the ability to recapitulate renal development and cellular diversity. Nevertheless, challenges related to their maturity, variability, and complexity persist. Continued research and advancements in this field are expected to address these limitations and enhance the utility of organoids for nephrotoxicity studies and applications in regenerative medicine.

## Methods of obtaining human kidney cells

Renal Biopsy: This procedure entails inserting a needle into the kidney to obtain a small tissue sample. While it can provide valuable insights into kidney health, it is invasive and carries potential risks such as bleeding, infection, and kidney damage. Renal biopsy is primarily used for diagnostic purposes rather than routine research due to its invasiveness and cost, which can vary depending on the location and medical facility [63].

Kidney Organ Donation: This method involves the removal of a kidney from a deceased or living donor. It offers a substantial number of kidney cells, making it advantageous for research purposes. However, it raises ethical considerations, particularly regarding the risk and benefit assessment for living donors [64]. The cost of kidney organ donation encompasses various expenses, including medical and surgical costs, and is relatively high.

Urinary Exfoliated Cell Isolation: This non-invasive approach entails collecting cells from patients' urine. It provides a large number of cells and has gained attention for its potential in research, particularly human urinary exfoliated cells (USCs), which have shown promise as a source of cells for drug-induced nephrotoxicity testing in 3D models. This method offers the advantage of being non-invasive, making it more acceptable for routine research and potentially reducing costs compared to invasive procedures [6–9].

In summary, the choice of method for obtaining kidney cells depends on the research goals, ethical considerations, invasiveness, and cost factors. Urinary exfoliated cell isolation, in particular, has emerged as a non-invasive and promising approach for research purposes, potentially overcoming some of the limitations associated with invasive procedures like renal biopsy or kidney organ donation.

## Personalized nephrotoxicity assessment using USC in 3D models

**Fig. 1:**
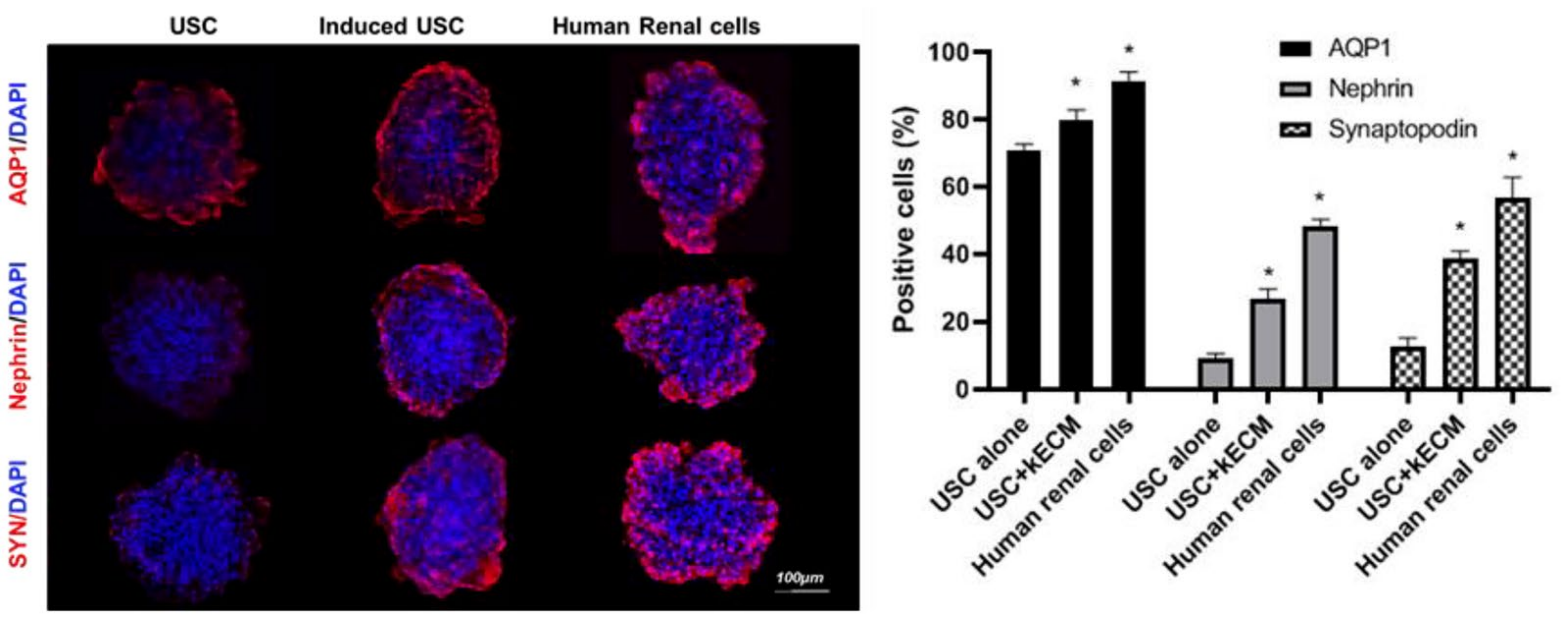
USC differentiated into renal cells in 3D culture 2 weeks after kidney extracellular induction Confocal images of 3D human USC organoids (scale bar, 100 μm) induced USC (4000 cells at p3) expressed renal tubular epithelial cell marker (AQP1) and podocyte markers (nephrin and synaptopodin, i.e. SYN); Quantification of cells expressing renal cell markers in 3D culture (n = 6). Data presented as mean ± SD. Significance: *p < 0.05

**Fig. 2:**
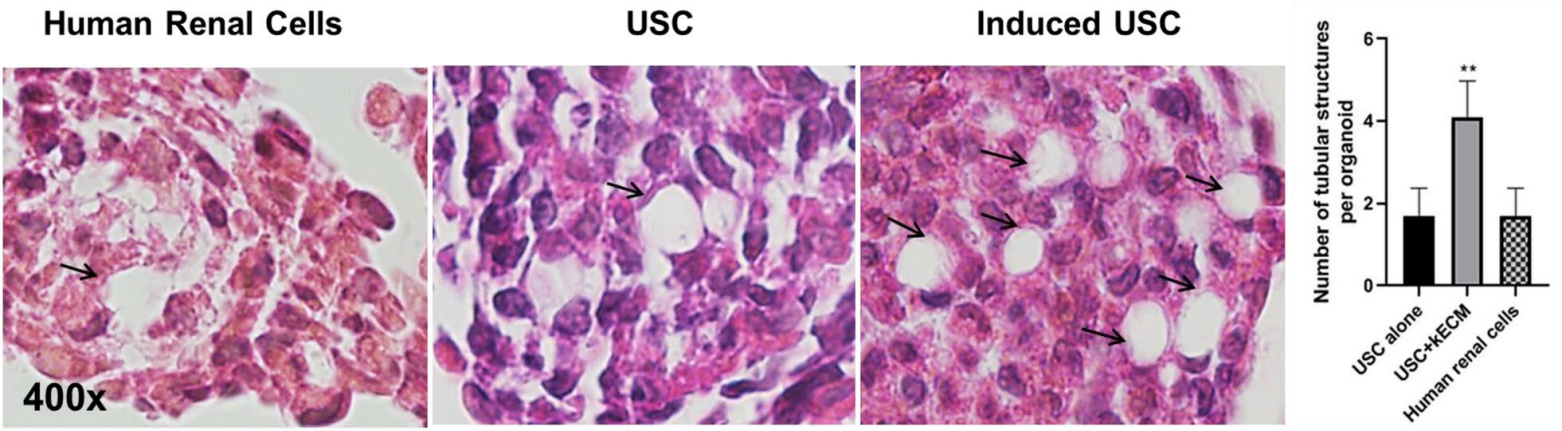
Tubular structure formation within organoids of USC two weeks after induced by kidney extracellular matrix in 3D organoids. Paraffin sections of USC organoids (arrows) stained by H&E (high magnification × 400). Quantification of tubular structure formation in 3D culture (n = 6). Data presented as mean ± SD. Significance: *p < 0.05

**Fig. 3:**
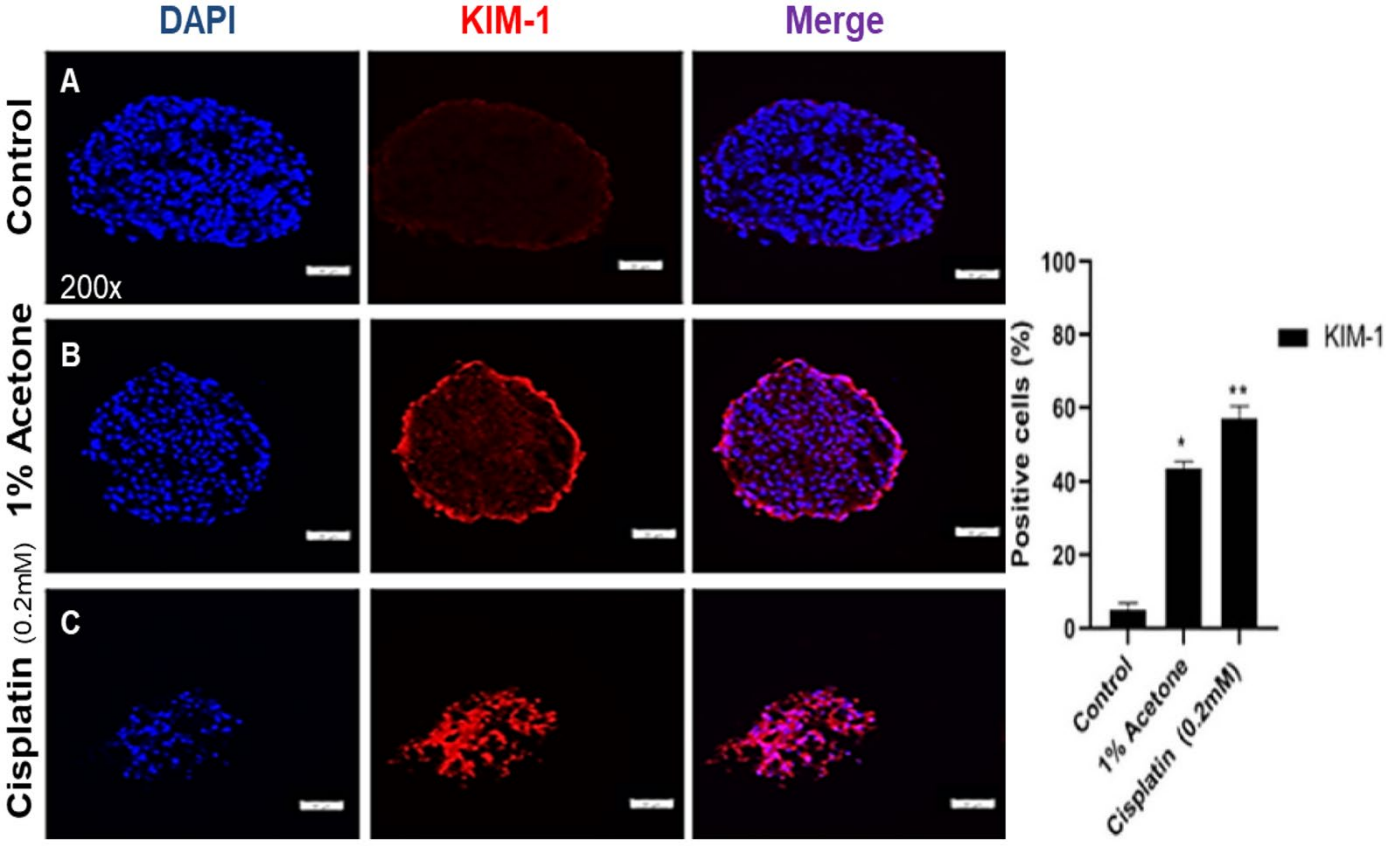
Drug-induced cytotoxicity assay on 3D kidney organoids with induced USC (p3). Three days after treatment with 1% acetone and cisplatin (200 μm/ml), approximately two-thirds of the cells exhibited apoptotic or necrotic cell death. Dead cells were subsequently washed away during media change, resulting in a visible loss of cells on the staining slide. Nephrotoxicity was assessed using Kidney Injury Molecule-1 (KIM1) levels after 3 days of exposure to cisplatin and acetone (n = 6). Data in graphs are expressed as mean ± SD. Immunofluorescence analysis using the R9 KIM-1 antibody (red) shows the expression of the target protein in USCs. The nuclei were stained with 4,6-diamino-2-phenylindole (DAPI, blue)

USCs are an easily accessible source of stem cells that can differentiate into various renal cell types including podocytes and renal tubular epithelial cells (Fig. 1), and form renal tubular structures in spheroid cultures (Fig. 2) [6]. USCs isolated from healthy individuals can be used to develop 3D models for testing drug induced renal toxicity (Fig. 3). We found that 3D models with USCs were able to predict the nephrotoxicity of several drugs, including gentamicin and cisplatin [6] and antiretroviral drug-induced renal mitochondrial toxicity [8, 9]. Additionally, USCs can be obtained from the elderly [65] and patients with renal diseases (such as renal tumors [66] and diabetic nephropathy [67]), making them a promising model for personalized nephrotoxicity testing. Thus, USCs are being investigated as a promising source of cells for the development of accurate and predictive 3D models for drug-induced nephrotoxicity testing and drug development.

**Fig. 4:**
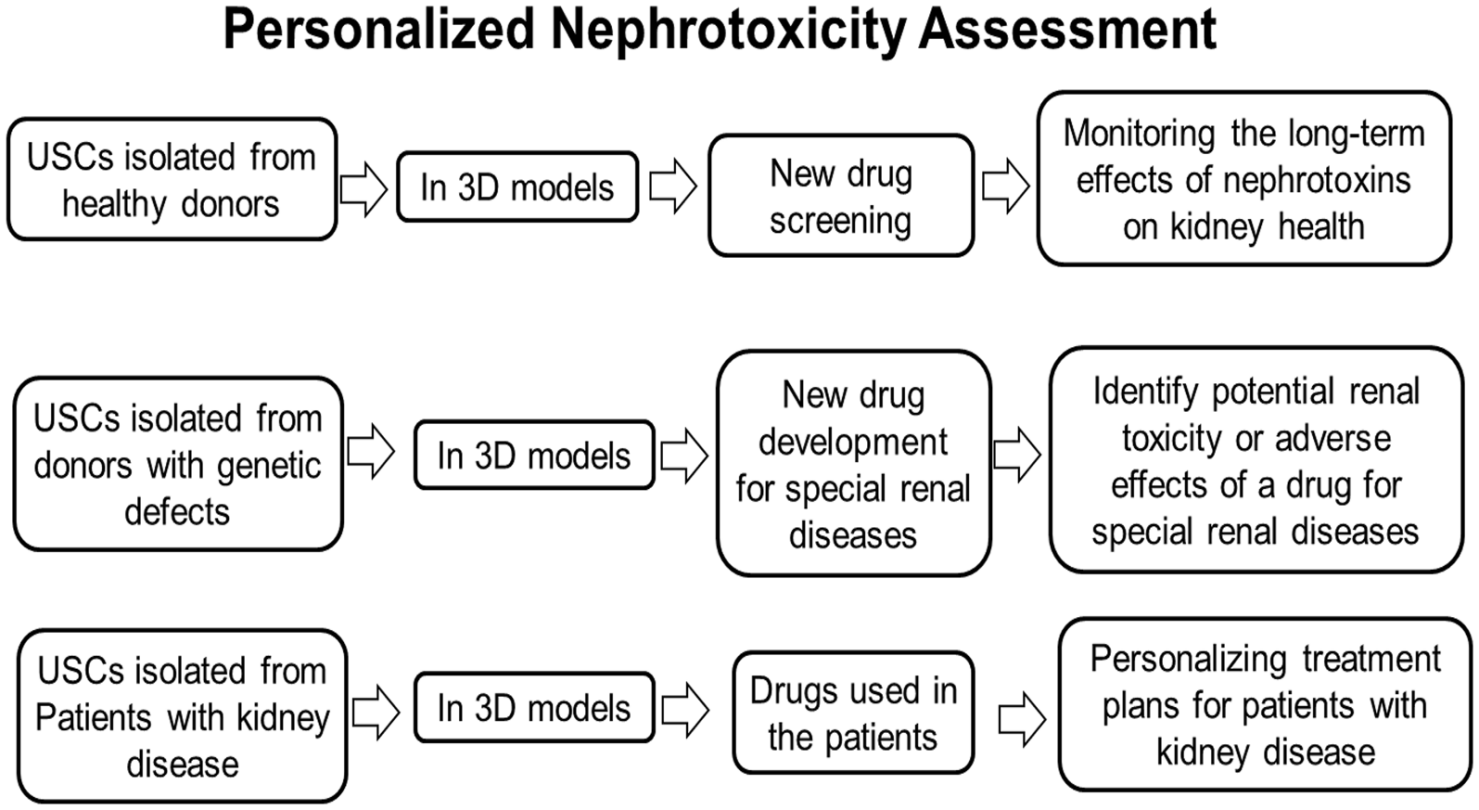
Personalized nephrotoxicity assessment in 3D models with human USCs

Personalized renal toxicology with USC in 3D culture models can involve two parts: drug development and drug-toxicity monitoring (Fig. 4):

Using USCs from healthy donors in 3D models for new drug screening: This approach involves testing the toxicity of a drug or compound using cells from healthy donors. By establishing a baseline of what is considered normal or safe, researchers can identify potential toxicities or adverse effects of a drug before it is tested on patients.

Using patients’ own USCs in 3D models for drug-toxicity monitoring: This approach involves testing the toxicity of a drug or compound using cells from patients. By testing autologous USCs, we can determine if a drug will be nephrotoxic or harmful to that individual. This can help personalize treatment plans and prevent adverse reactions/side effects. Importantly, USCs can be obtained from elderly donors, many of whom have reduced renal function, and patients with diabetic nephropathy to serve as biomarkers for aging and diabetes. Studies in our laboratory have found that USC from these patients USCs display weak or negative telomerase activity, higher levels of inflammatory factors (IL-1β and Cx43) and apoptotic markers (Caspase-3, and TUNEL), lower levels of autophagy markers (LC3-II) and mTOR signaling molecules (p-mTOR/mTOR, p-Raptor/Raptor and p-S6K1), less proliferation capacity (p < 8) and differentiation potential hindered by senescence as compared to USCs from healthy young donors [65, 67]. Despite having weak generation ability, USCs from these patients still possess normal stemness properties, and are sufficient in small numbers (3–5 USC clones/100 ml urine) to be used for 3D renal toxicity assessment [65, 67].

Both approaches can be valuable in drug development and clinical use: using USCs from healthy donors for new drug screening can help identify potential toxicities early in the drug development process, while using patient cells for drug-toxicity monitoring can help personalize treatment plans. However, personalized toxicology can be time-consuming and expensive, and may not always be feasible for large-scale drug development or clinical use.

## 3D bioprinting for drug-induced nephrotoxicity testing

Advances in 3D bioprinting solid organs have been reviewed recently [68]. 3D printing has also been explored to create 3D structures for testing drug-induced nephrotoxicity. This method involves using a printer-like device to deposit a bioink made of living cells, growth factors and other biologically relevant materials layer-by-layer to create the desired renal structure. It allows for precise control over the architecture and geometry of the structure and can be used to construct complex and heterogeneous structures for drug testing.

Numerous studies have utilized 3D printing to develop 3D cultures for testing drug-induced nephrotoxicity. For example, Homan et al. used 3D printing to generate a kidney-on-a-chip device that could be used for drug screening [34]. Another study used 3D printing to create a 3D cell culture system that could be used to examine the nephrotoxicity of cisplatin [35]. In summary, 3D printing offers several advantages: it allows for the creation of complex structures with precise control over their size and shape, can even be used to develop personalized models for testing drug-induced nephrotoxicity.

## Microfabrication for 3D cultures

Microfabrication for 3D cultures refers to the use of advanced microfabrication techniques to create complex three-dimensional (3D) structures and environments for culturing cells and tissues in a laboratory setting. Microfabrication for 3D cultures and 3D bioprinting are both powerful tools for tissue engineering, but they have different approaches and attributes [69, 70]. 3D bioprinting allows for the direct printing of living cells and the creation of structures with high control and precision, while microfabrication is ideal for creating complex structures with precise spatial organization of cells and the extracellular matrix [7, 20, 35, 38, 49, 71, 72]. Particularly, microfabrication techniques allow for the precise control of the structure's size, shape, and spatial organization of cells within the structure. Microfabrication for 3D cultures typically uses biocompatible materials such as hydrogels (e.g. collagen [73]) or polymer scaffolds [8] to create the structure of renal tissue. This technique is ideal for creating structures with human renal cells [8] for drug induced nephrotoxicity testing, such as organs-on-chips or microphysiological systems.

We have used microfabrication methods to developed a 3D cell platform using human primary USCs in a silk fiber matrix (SFM) to predict drug-induced mitochondrial toxicity [8], and compared this approach to USC grown in spheroids. The results showed that USC numbers remained steady in both 3D spheroids and SFM, but up to 95% of USC survived in 3D SFM, while cell numbers significantly declined in 3D spheroids after six weeks. The highly porous SFM provided a large number of cells, enabling multiple experiments with less labor and lower cost compared to 3D spheroids. The levels of mtDNA content, mitochondrial superoxide dismutase2 (SOD2) as an oxidative stress biomarker, and cell senescence genes of USC were stably retained in 3D USC-SFM, while significantly increasing over time in spheroids. Both 3D culture models showed a significant decrease in mtDNA content and mitochondrial mass six weeks after treatment with the mitotoxic antiretroviral drug 2′-3′-Dideoxycytidine (ddC). However, levels of complexes I, II, and III significantly decreased in 3D SFM-USC treated with ddC, compared to only complex I level in spheroids. A dose- and time-dependent chronic MtT was displayed in the 3D USC-SFM model, but not in spheroids. Thus, a long-term 3D SFM culture model of human primary USC is a reliable, cost-effective, and sensitive approach for the assessment of drug-induced chronic mitochondrial toxicity.

Several other studies have also utilized microfabrication to create 3D cultures for drug-induced nephrotoxicity testing. For example, one study [30] developed a microfluidic device for drug screening with renal tubules. The use of a microfluidic platform is an innovative approach to replicate the fluid transport functions of renal cells. Through this platform, renal epithelial cells can actively generate hydraulic pressure gradients, which decline with increasing hydraulic pressure until reaching a stall pressure, similar to mechanical fluid pumps. For normal human kidney cells, the direction of fluidic flux is from apical to basal, and the pressure is higher on the basal side. Therefore, mechanical force and hydraulic pressure are essential factors in kidney function and morphological changes. This platform provides valuable insights into the physiological or pathophysiological mechanisms involved in the development and transduction of hydraulic pressure gradients, paving the way for improved understanding and treatment of kidney-related diseases and for drug toxicology [30]. Another study used microfabrication to create a 3D culture system that included a microfluidic chip and a collagen-based scaffold to examine the nephrotoxicity of cisplatin [29, 74].

To summarize, microfabrication has been used to create 3D cultures for drug-induced nephrotoxicity testing. Microfabrication offers several advantages, including precise control over the cellular microenvironment and the creation of complex structures and high throughput systems for drug screening.

## A list of commonly used drugs for nephrotoxicity testing

Some commonly used drugs for nephrotoxicity testing include aminoglycosides, cisplatin, NSAIDs, and antimicrobials (Table 4). Most of these drugs cause renal toxicity by reducing ATP generation, DNA, mitochondria function, and inducing oxidative stress, and cell apoptosis [75, 76] Based on tissue injury type and clinical presentation, antimicrobial-induced nephropathy may result in acute tubular necrosis, acute interstitial nephritis and crystal (obstructive) nephropathy.

**Table 4:** A list of commonly used drugs for nephrotoxicity testing

Drug	Class	Treatment	Targeted renal cells	Mechanism of renal injury/Outcomes	Doses	Refs.
Gentamicin	Aminoglycoside antibiotics	Bacterial infections	Proximal tubular cells	Acute tubular necrosis from direct cytotoxicity	100 mg/kg	[77, 78]
Vancomycin	Glycopeptide antibiotics	Bacterial infections	Proximal tubule cells	Acute tubular necrosis following oxidative stress and (most commonly) and tubular cast formation	200 mg/kg	[79–81]
Tenofovir	Antiretroviral drug	HIV/HBV infection	Renal tubule cells and glomerulus	Tubular dysfunction and mitTox	300 mg/kg	[82–84]
Amphotericin B	Antifungal medication		Distal tubule and smooth muscle cells	Membrane permeability and vaso-constriction	4 mg/kg	[85, 86]
Cisplatin	Chemotherapy	Various types of cancers	Renal proximal tubule	Inflammation, apoptosis, oxidative stress, dna damage, & mitotox	20 mg/kg	[87, 88]
Methotrexate	Chemotherapy	Various types of cancers, autoimmune diseases	Proximal tubule	Apoptosis	20 mg/kg	[89–91]
Cyclosporine	Immunosuppressive drug	Prevent organ transplant rejection, autoimmune diseases	Proximal tubules	Oxidative stress, Tubular necrosis	25 mg/Kg	[92, 93]
Acetaminophen	Over-the-counter	Pain reliever and fever reducer	Proximal tubule	Acute tubular necrosis	500 mg/kg	[94, 95]
Ibuprofen	Over-the-counter NSAID	Relieve pain and inflammation	Tubular cell epithelial cell	Interstitial nephritis, transitional tubular necrosis	400 mg/kg	[96, 97]
Lithium	Anti-mania	Bipolar disorder	Distal tubule and proximal tubular	ROS formation, Lipid, per-oxidation, and antioxidant Mechanisms	50 mg/kg	[98, 99]

*MitTox* mitochondrial toxicity, *NSAID* nonsteroidal anti-inflammatory drug, *ROS* radical oxygen species

These drugs are commonly used in nephrotoxicity testing because they have been shown to cause kidney damage in certain situations or at high doses. However, it is important to note that not all individuals will experience nephrotoxicity from these drugs, and the severity of nephrotoxicity can vary depending on factors such as dose, duration of use and individual susceptibility. Other drugs or compounds may also be tested for nephrotoxicity, depending on the specific research question or application.

## Commonly used parameters for nephrotoxicity testing

Some commonly used parameters for nephrotoxicity testing include cell viability, mitochondrial function, reactive oxygen species production, and inflammatory markers (Table 5):

**Table 5:** A list of commonly used parameters for nephrotoxicity testing

Assessment	Measurements
Cell viability assays	Degree of cell death or damage in response to a drug or other compound, which can indicate potential NT assessed CCK-8, MTT and live/death kits
Biomarker analysis	Expression of biomarkers associated with kidney function (e.g., albumin, nephrin) for the effects of a drug or compound on kidney cells
Histological analysis	3D kidney tissue constructs under a microscope can reveal structural changes indicative of kidney damage
OCR/ECAR	Metabolic parameters can provide information on cellular respiration and glycolysis, which can be affected by nephrotoxic compounds, seahorse analysis
ROS	ROS levels can provide insight into this aspect of nephrotoxicity
Inflammatory markers	Cytokines genes and proteins: TNF-α, IL-1β, IL-6, IL-8, MCP-1, HMGB1, CRP, PGE2 and NO, assessed by q-PCR and western-blot
Kidney-specific protein markers	Expression of proteins: e.g., aquaporins (AOP1, AQP3), transporters (i.e., OAT1, OAT3, OCT2, MRP2, BCRP, SGLT2)
Mitotoxicity	MMP, ATP production, complexes I-V expression and activity, mitochondrial morphology, mtDNA content and OCR/ECAR

*OCR* oxygen consumption rate, *ECAR* extracellular acidification rate, *ROS* reactive oxygen species assays, *MMP* Mitochondrial membrane potential, *ATP* adenosine triphosphate, *mtDNA* content-mitochondrial DNA content

The specific parameters used to assay nephrotoxicity in 3D culture depend on the research question and the drug being tested. It is important to note that no single parameter is sufficient to fully assess nephrotoxicity, and a combination of assays is frequently used to gain a more comprehensive understanding of the potential for kidney damage.

Several inflammatory markers are used to assess nephrotoxicity in 3D in vitro models with renal and immune cells. They include: (1) Tumor necrosis factor-alpha (TNF-α): TNF-α is a pro-inflammatory cytokine produced by various cells, including macrophages and monocytes. It plays a critical role in the immune response and is a potent inducer of inflammation; (2) Interleukin-1 beta (IL-1β): IL-1β is another pro-inflammatory cytokine produced by monocytes, macrophages, and other cells. It is involved in the regulation of the immune response and the induction of fever; (3) Interleukin-6 (IL-6): IL-6 is a cytokine produced by T cells, B cells, and macrophages. It plays a critical role in the immune response and is a potent inducer of inflammation; (4) Interleukin-8 (IL-8): IL-8 is a chemokine produced by epithelial cells and macrophages. It plays a critical role in the recruitment of neutrophils to sites of inflammation; (5) Monocyte chemoattractant protein-1 (MCP-1): MCP-1 is a chemokine produced by monocytes, macrophages, and endothelial cells. It plays a critical role in the recruitment of monocytes to sites of inflammation; (6) High-mobility group box 1 (HMGB1): HMGB1 is a nuclear protein released by necrotic cells and acts as a damage-associated molecular pattern (DAMP) molecule. It can activate the immune system and induce inflammation; (7) C-reactive protein (CRP): CRP is an acute-phase protein produced by the liver in response to inflammation. It can be used as a biomarker of inflammation; (8) Prostaglandin E2 (PGE2): PGE2 is a lipid mediator produced by various cells, including macrophages and epithelial cells. It plays a critical role in the regulation of inflammation and pain; (9) Nitric oxide (NO): NO is a free radical produced by various cells, including macrophages and endothelial cells. It plays a critical role in the regulation of inflammation and the immune response.

In addition, Several transporters expressed in the proximal tubules of the kidney are also used to assess nephrotoxicity in vitro, including: (1) Organic anion transporters 1 and 3 (OAT1, OAT3): OAT1 and OAT3 play a critical role in the transportation of organic anions, including some drugs and toxins; (2) Organic cation transporter 2 (OCT2): OCT2 plays a critical role in the transport of organic cations; (3) Multidrug resistance protein 2 (MRP2): MRP2 plays a critical role in the transportation of conjugated organic anions; (4) Breast cancer resistance protein (BCRP): BCRP plays a critical role in the transportation of organic anions and cations; (5) Sodium-glucose cotransporter 2 (SGLT2): SGLT2plays a critical role in the reabsorption of glucose from the filtrate; (6) Aquaporins: Aquaporins are a family of water channels expressed in various parts of the kidney, including the proximal tubules, and play a critical role in the reabsorption of water. Other transporters can also be used,depending on the relevance to the specific mechanism of nephrotoxicity being studied.

## Exploring the mechanisms of drug-induced precision nephrotoxicity

Drug-induced precision nephrotoxicity is characterized by alterations in the expression of mRNAs and miRNAs in human primary renal cells or patient-derived renal cells (i.e., USCs), which can influence the development and progression of the disease, as well as play protective roles [100]. The regulation of the transcriptome is modulated through a variety of pathways, including transcriptional initiation, RNA processing and post-translational modification, which can affect the expression of genes involved in the pathological or recovery processes of drug induced kidney disease (DIKD). miRNA plays an important role in regularizing drug metabolization and transportation, and is a potentially promising proxy marker to assess drug efficacy and safety [101].

Specifically, circulating miRNA levels regulate the gene activity when tissues are exposed to toxic substances, making them novel non-invasive and sensitive biomarkers for drug-induced tissue injury pathologies [102]. miRNAs also contribute to renal toxicity by modulating the expression of downstream target genes that can either promote renal protection or exacerbate the disease. By deciphering the interplay between changes in the transcriptome and miRNA expression, it may be possible to facilitate early diagnosis, develop innovative therapies, and evaluate the prognosis of patients with nephrotoxicity. Although a promising tool to understand the mechanism of toxicology, challenges still exist in standardizing the analysis of the miRNA data, which need to be addressed appropriately to facilitate the successful translation into clinical practice [103].

Genomics, transcriptomics in particular can be very helpful in exploring the mechanism of drug-induced precision nephrotoxicity in 3D in vitro renal models using human autologous renal cells. By analyzing gene expression patterns in response to a drug treatment, transcriptomics can provide valuable insights into the molecular mechanisms underlying drug-induced nephrotoxicity. For example, RNA-seq analysis can be used to quantify changes in gene expression levels in response to a drug treatment [29]. This can help identify specific genes and pathways that are affected by the drug, providing clues as to how the drug is causing nephrotoxicity. Additionally, other analyses such as epigenomics or proteomics can be used to explore changes in DNA methylation patterns, histone modifications or protein expression in response to a drug treatment. Together, these techniques can help identify potential biomarkers of nephrotoxicity, which could be used to develop more sensitive and specific assays for predicting and monitoring drug-induced nephrotoxicity in vitro and in vivo.

In summary, genomics can play an important role in elucidating the mechanisms of drug-induced precision nephrotoxicity in 3D in vitro renal models using human primary USCs or patients own renal cells and help develop new strategies for the prevention and treatment of drug-induced kidney damage.

## Conclusions

The use of 3D in vitro models with human primary renal cells can help reduce the dependence on animal models for drug testing. Compared to 2D models, 3D models are more ethical, cost-effective, and predictive.

- 3D cultures can better mimic the complex microenvironment of the kidney, including cell–cell interactions, extracellular matrix deposition and oxygen and nutrient gradients, leading to more physiologically relevant results.
- Various techniques can be used to create 3D cultures for nephrotoxicity testing, such as 3D printing, microfabrication, and bio-printing. These techniques allow for precise control over the structure and composition of the 3D cultures, leading to reproducible and consistent results.
- Human primary culture cells are generally considered the best choice for 3D cultures, although animal renal cells and cell lines are also used. USCs are a promising option for generating 3D kidney tissue constructs. However, further research is needed to validate their suitability for nephrotoxicity testing.
- Commonly used drugs for nephrotoxicity testing include antibiotics, chemotherapeutic agents, and nonsteroidal anti-inflammatory drugs (NSAIDs). Several parameters can be used to assay nephrotoxicity in 3D cultures, including cell viability, biomarker analysis, histological analysis, metabolic parameters, ROS assays, inflammatory markers, and kidney-specific protein expression analysis.

In conclusion, in vitro models for precision nephrotoxicity testing using 3D cultures of patient-derived renal cells hold great promise for improving drug safety and reducing the need for animal testing. However, further validation and standardization of these models are needed to ensure their widespread use and acceptance in the pharmaceutical industry.

## Data Availability

Not applicable.

## Abbreviations

OCR/ECAR: Oxygen consumption rate/extracellular acidification rate
MMP: Mitochondrial membrane potential
ATP: Adenosine triphosphate
mtDNA: Mitochondrial DNA
ROS: Reactive oxygen species
USCs: Urine-derived stem cells
2/3D: Two/Three-dimensional
iPSC: Pluripotent stem cells
PCL: Polycaprolactone
PCL: Poly (lactic-co-glycolic acid)
SYN: Synaptopodin
KIM1: Kidney injury molecular-1
SFM: Silk fiber matrix
SOD2: Superoxide dismutase2
ddC: 2′-3′-Dideoxycytidine
MtT: Mitochondrial toxicity
TNF-α: Tumor necrosis factor-alpha
IL-1β: Interleukin-1 beta
IL-6: Interleukin-6
IL-8: Interleukin-8
MCP-1: Monocyte chemoattractant protein-1
HMGB1: High-mobility group box 1
DAMP: Damage-associated molecular pattern
CRP: C-reactive protein
PGE2: Prostaglandin E2
NO: Nitric oxide
OAT1: Organic anion transporter 1
OAT3: Organic anion transporter 3
OCT2: Organic cation transporter 2
MRP2: Multidrug resistance protein 2
BCRP: Breast cancer resistance protein
SGLT2: Sodium-glucose cotransporter 2
NSAIDs: Nonsteroidal anti-inflammatory drugs
DIKD: Drug induced kidney disease
